# Rising temperatures may drive fishing-induced selection of low-performance phenotypes

**DOI:** 10.1038/srep40571

**Published:** 2017-01-17

**Authors:** Timothy D. Clark, Vanessa Messmer, Andrew J. Tobin, Andrew S. Hoey, Morgan S. Pratchett

**Affiliations:** 1 Australian Institute of Marine Science, PMB 3, Townsville MC, Townsville, Queensland, Australia; 2 ARC Centre of Excellence for Coral Reef Studies, James Cook University, Townsville, Australia; 3 College of Marine and Environmental Sciences and Centre for Sustainable Tropical Fisheries and Aquaculture, James Cook University, Townsville, Australia

## Abstract

Climate warming is likely to interact with other stressors to challenge the physiological capacities and survival of phenotypes within populations. This may be especially true for the billions of fishes per year that undergo vigorous exercise prior to escaping or being intentionally released from fishing gear. Using adult coral grouper (*Plectropomus leopardus*), an important fisheries species throughout the Indo-Pacific, we show that population-level survival following vigorous exercise is increasingly compromised as temperatures increase from current-day levels (100–67% survival at 24–30 °C) to those projected for the end of the century (42% survival at 33 °C). Intriguingly, we demonstrate that high-performance individuals take longer to recover to a resting metabolic state and subsequently have lower survival in warm water compared with conspecifics that exercise less vigorously. Moreover, we show that post-exercise mortality of high-performance phenotypes manifests after 3–13 d at the current summer maximum (30 °C), while mortality at 33 °C occurs within 1.8–14.9 h. We propose that wild populations in a warming climate may become skewed towards low-performance phenotypes with ramifications for predator-prey interactions and community dynamics. Our findings highlight the susceptibility of phenotypic diversity to fishing activities and demonstrate a mechanism that may contribute to fishing-induced evolution in the face of ongoing climate change.

Anthropogenic carbon emissions and modified land use have directly contributed to increases in the surface temperature of the planet since the beginning of the industrial revolution^1^. Aquatic systems have absorbed the majority of the excess heat added to the atmosphere, which has led to a warming of the global sea surface by 0.4 °C in the past century with an additional 0.6–2.0 °C expected by 2100^1^,^2^. Concomitant with the background warming, there is evidence that extreme shorter-term temperature spikes (e.g., daily, seasonal) are becoming more frequent in many aquatic systems, presenting a more immediate thermal challenge for aquatic organisms^1^,^3^,^4^,^5^. While the direct effects of environmental warming on the survival, ecology and physiology of aquatic organisms are becoming clearer^3^,^6^,^7^,^8^, it is generally accepted that other stressors can work interactively with temperature to increase stress and reduce survival^9^,^10^. The future performance of aquatic organisms is therefore dependent upon the combined impacts of multiple stressors working simultaneously as the environment continues to change.

Exercise is critical for optimising fitness through processes such as food capture, predator avoidance and reproduction. Nevertheless, exercise often induces physiological, biochemical and behavioural disturbances that require significant durations to recover to baseline levels^11^,^12^,^13^,^14^,^15^. Thus, the interplay between climate warming and exercise is a critical consideration in helping to understand long-term climate impacts on animal populations, yet one that has received scant attention. Fishes differ greatly in their capacity for exercise and recovery, from high-performance athletes that swim continuously in the open ocean (e.g., tunas), to ambush predators that undergo short periods of burst swimming interspersed with long periods of inactivity (e.g., flatfishes). As such, the physiological and biochemical systems of fishes have adapted to cope with different levels of exercise stress encountered throughout their lifecycle. Anthropogenic influences can elicit a level of exercise in fishes that far exceeds natural levels. For fishes that undergo vigorous exercise during fisheries capture but are subsequently released (e.g., incidental bycatch, undersized individuals, catch-and-release recreational captures^16^,^17^), more than 80% of individuals may ultimately die^18^,^19^,^20^. It has been estimated based on Canadian statistics that billions of fishes may be caught and released globally each year in the recreational sector alone^21^, emphasising the critical importance of this driver in the management and conservation of global fish populations. While there is some evidence that thermal conditions can influence the magnitude of post-exercise mortality within fish species^18^,^22^,^23^,^24^, it is poorly understood how phenotypic differences in exercise propensity may contribute to fishing-induced selection or evolution in a warming world^25^,^26^,^27^,^28^,^29^.

Although the underlying mechanisms responsible for post-exercise mortality remain speculative^24^,^30^, one of the leading hypotheses to explain the performance and survival of fishes across temperature is termed ‘oxygen- and capacity-limited thermal tolerance’ (OCLTT)^31^,^32^. While highly contentious^33^, this hypothesis proposes that performance and survival across temperature is universally governed by the capacity to increase oxygen transport from baseline levels (resting metabolic rate, RMR) to maximum levels (maximum metabolic rate, MMR). The temperature-dependent difference between RMR and MMR, termed aerobic scope (AS), is thought to increase to a peak at an ecologically optimal temperature and then decline progressively or abruptly as temperature continues to increase towards lethal levels. Accordingly, it may be expected based on OCLTT that a temperature-induced decline in aerobic scope would be responsible for any increase in post-exercise mortality and thus contribute to fishing-induced selection at high temperatures.

Coral reef organisms have evolved in some of the most thermally stable aquatic environments on the planet and thus may be particularly susceptible to forecasted climate warming^4^,^6^,^34^,^35^. Many coral reef fishes are targeted by commercial and recreational fisheries (e.g., hook-and-line or net capture), with a significant portion of the catch returned to the water following capture. The leopard coral grouper (*Plectropomus leopardus*) is a prized fisheries species throughout the Indo-Pacific^36^,^37^, where it encounters temperatures ranging ~20–31 °C across its latitudinal distribution. Approximately 1,350 tonnes of coral grouper are caught and retained annually by the commercial sector on the Great Barrier Reef (GBR) alone (35% of the total line catch). The number of coral grouper caught-and-released annually by the Australian recreational sector is unknown, although it is likely to be at least as high as the estimated commercial fishery discard rates (200,000–600,000 fish per year^38^). Contemporary management regulations in Australia mandate the return of coral grouper to the water if total length is under 38 cm, if the bag limit of seven fish per recreational fishing boat has been reached, or if commercial vessels reach their quota allocation for the species^36^. Thus, important fisheries species like the coral grouper will increasingly face the combined challenges of climate warming and fishing-induced exercise stress.

Using coral grouper as a model species, here we investigate how climate warming may interact with exercise stress to contribute to fishing-induced selection. Coral grouper are site-attached piscivores that rarely undergo sustained exercise of more than a few seconds when, for example, they burst to capture prey or retreat under coral cover to avoid a passing shark^39^,^40^. In contrast, fishing-related stressors for coral grouper can include several minutes of exhaustive exercise during hook-and-line capture (particularly in the recreational sector), and a period of air exposure prior to the fish being released back to the water. In fact, a catch-and-release fishing event is likely to constitute the most extreme exercise stress that a coral grouper will experience in its lifetime, emphasising the potential importance of exercise propensity in contributing to fishing-induced selection. Employing standardised exercise protocols and acclimation groups that span most of the current and forecasted (to 2100) temperature range of the GBR, we use measurements of metabolic rate and long-term survival to understand the exercise and recovery performance of coral grouper across current and forecasted thermal conditions. We hypothesise that impaired oxygen transport during recovery progressively compromises the post-exercise survival of particular phenotypes as temperatures approach those predicted to occur on the GBR by the end of the century. Importantly, we assess subsets of the same individuals after 2–3 weeks of thermal acclimation (‘early acclimated’) and again after 5–6 weeks of thermal acclimation (‘fully acclimated’) to quantify the temporal dynamics of metabolic acclimation and the repeatability of our exercise performance scoring system (1 through 5.5) associated with our fisheries capture simulation.

## Results

**Figure 1:**
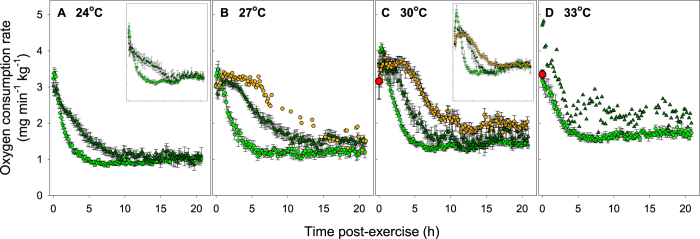
Recovery in the oxygen consumption rate (means ± SE) of coral grouper following the burst-exercise fishing simulation in fully-acclimated fish (i.e., ~6 weeks) at (**A**) 24 °C, (**B**) 27 °C, (**C**) 30 °C, and (**D**) 33 °C. Insets in (**A**) and (**C**) show data for early-acclimated fish (i.e., 2-3 weeks). Survivors are separated into fish that achieved exercise performance scores of ≤3 (light green circles) and those with scores >3 (dark green triangles). Delayed mortalities are presented as orange circles. Short-term mortalities died within respirometers and thus only the first post-exercise measurement was taken for each fish (large red circles). Sample sizes given in Fig. 3.

**Figure 2:**
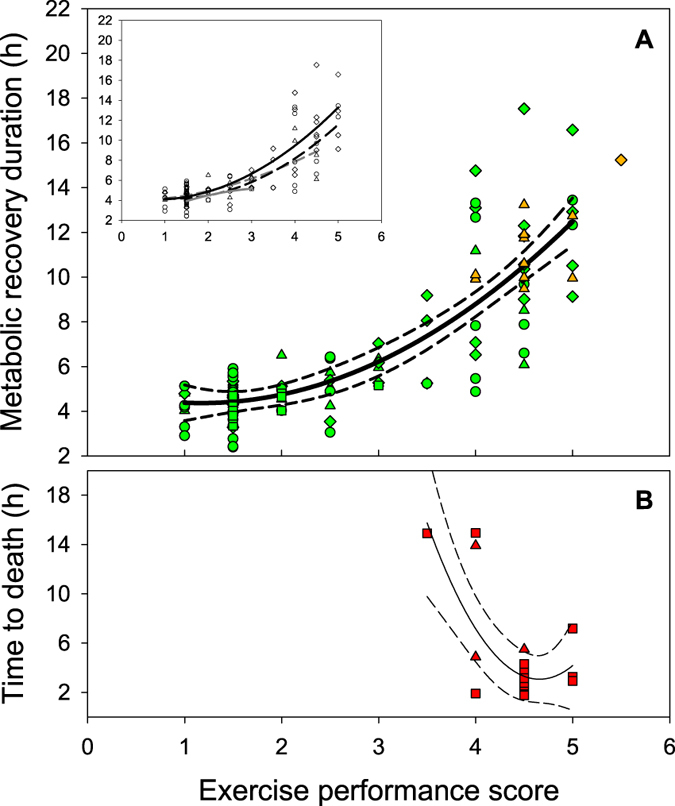
Metabolic recovery duration of survivors and delayed mortalities (**A**) and time to death of short-term mortalities (**B**) of coral grouper as a function of the exercise performance score obtained during the fishing simulation, where circles represent fish acclimated to 24 °C, diamonds represent fish acclimated to 27 °C, triangles represent fish acclimated to 30 °C, and squares represent fish acclimated to 33 °C. Inset in (**A**) shows the regressions for the survivors at each temperature, but there were no differences between temperatures (see text) so an overall regression is presented in the main panel of (**A**) with 95% confidence bands (regression applies only to survivors [green], but delayed mortalities from both the early-acclimated and fully-acclimated groups are displayed for comparative purposes [orange]). Short-term mortalities (red) occurred only in fish at 30 and 33 °C and only when exercise performance scores were 3.5 or above.

The fishing simulation (3 min burst exercise +1 min air exposure) caused a large elevation in oxygen consumption rate (O_2_) that gradually declined to resting levels (i.e., RMR) over 2–18 hours in both the early-acclimated and fully-acclimated groups (Fig. 1). Differences in recovery duration were strongly dependent on exercise performance scores (*F*_(8,97)_ = 29.91, P < 0.001) but were not dependent on temperature (*F*_(1,97)_ = 0.185, P = 0.668; one-way ANCOVA on ‘survivors’; Fig. 2). Fish achieving higher exercise scores took longer to recover (Fig. 2), which is clearly illustrated when data from survivors are pooled into exercise scores of ≤3 (relatively poor performers; green circles Fig. 1) versus >3 (relatively good performers; green triangles Fig. 1). Importantly, there was evidence that exercise performance scores were repeatable for individuals between the early-acclimation and the full-acclimation trials (Table 1). Spearman correlation analysis highlighted strong repeatability of performance scores in the individuals at 24 °C (correlation coefficient (*r*_s_) = 0.797; P < 0.0001; N = 16), but a similar trend at 30 °C did not reach statistical significance, probably due to lower sample sizes and a lower range of performance scores resulting from delayed mortalities (occurring within 3–13 d) in the early-acclimation trials (*r*_s_ = 0.390; P = 0.197; N = 12) (Table 1).

As expected, RMR increased consistently with temperature between 24 and 30 °C in the early-acclimation experiments (Fig. 1 insets; t-test: t_(26)_ = −5.443, P < 0.001) and across 24–33 °C in the fully-acclimated animals (Fig. 1, Table 2; one-way ANOVA: *F*_(3,103)_ = 31.4, P < 0.001). RMR was higher in the survivors from the 24 °C early-acclimation group compared with the same individuals once they were fully-acclimated (0.93 ± 0.06 vs. 0.72 ± 0.05 mg min^−1^ kg^−1^, respectively; paired t-test: t_(15)_ = 3.801, P = 0.002; Fig. 1A, Table 1). The RMR of these individuals after full acclimation was not different from the other fish in the fully-acclimated treatment group at 24 °C (0.74 ± 0.04 mg min^−1^ kg^−1^; t-test: t_(37)_ = −0.329, P = 0.744), suggesting that the observed decrease in RMR over time was due to continued thermal acclimation and not due to greater familiarity with the respirometers. A trend of RMR decreasing over time in the 30 °C individuals used in both the early-acclimated and fully-acclimated experiments was also present (1.53 ± 0.08 vs. 1.24 ± 0.05 mg min^−1^ kg^−1^, respectively), but was not statistically significant (paired t-test: t_(11)_ = 1.762, P = 0.106). No other measured variables differed at either of the two temperatures between the early-acclimated trials and the trials on the same individuals after full acclimation.

**Figure 3:**
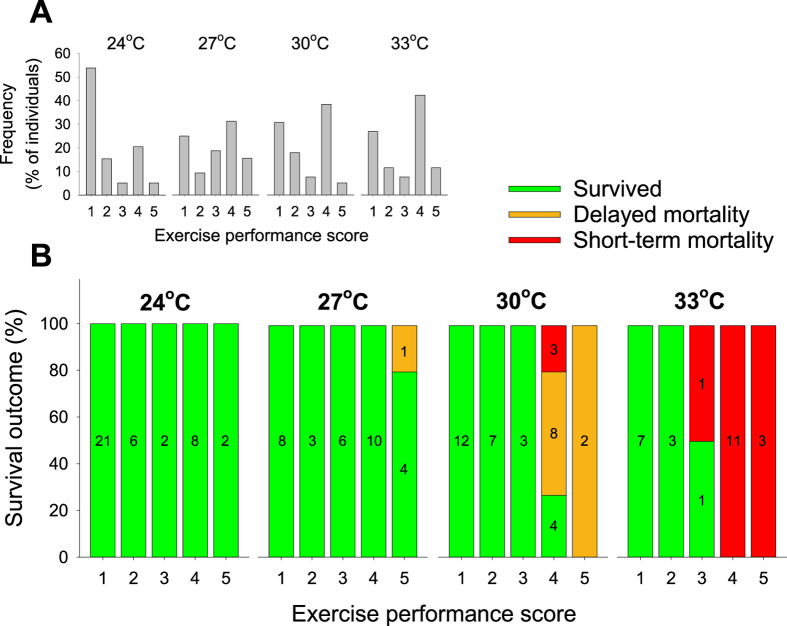
Frequency distributions of exercise performance scores (**A**) and survival outcomes as a function of exercise performance scores (**B**) of coral grouper in the four temperature treatment groups. Green, orange and red colouration represents survivors, delayed mortalities and short-term mortalities, respectively. Sample sizes are given in the bars of (**B**). For visual clarity, exercise performance scores were rounded down such that a score of 3.5 became 3.0, etc. For individuals that were tested in the early-acclimation group as well as following full acclimation (subsets in the 24 and 30 °C groups; Table 1), the trial after full thermal acclimation is the one included here. However, four fish from the early-acclimation group at 30 °C had exercise performance scores ≥4 and suffered delayed mortality (Table 1), and thus are included here.

The distribution of exercise performance scores was generally bimodal in each of the fully-acclimated groups, whereby the vast majority of fish achieved a score of 1.0–1.5 or 4.0–4.5 (Fig. 3A; scores binned into increments of 1 for clarity). The proportion of fish achieving exercise scores of 4.0–4.5 decreased dramatically at the lowest acclimation temperature of 24 °C, perhaps suggestive of a thermal threshold for muscle contraction frequencies (Fig. 3A).

Survival following the fishing simulation was negatively influenced by temperature in a non-linear fashion (100% at 24 °C, 97% at 27 °C, 67% at 30 °C, 42% at 33 °C; Fig. 3B). Interestingly, the exercise scores provided valuable insight into the survival patterns, revealing that survival was not only temperature-dependent, but also strongly dependent on the propensity for burst swimming at the level of the individual. That is, individuals that achieved high performance scores during the fishing simulation had a much greater probability of subsequent mortality (e.g., only 12% of fish (4 out of 33) survived after achieving an exercise score ≥4 at 30–33 °C). The rate of mortality was also temperature-dependent, with delayed mortalities accounting for the single death at 27 °C and the majority of deaths at 30 °C, while deaths at 33 °C were exclusively short-term mortalities (i.e., within 20 h; Fig. 3B). On average, the individuals that suffered short-term mortality died in the respirometers after 8.1 ± 2.9 h (range 4.9–13.9 h) in the 30 °C treatment (N = 3) and after 4.8 ± 1.1 h (1.8–14.9 h) in the 33 °C treatment (N = 15; Fig. 2B).

Fish suffering delayed mortality were almost exclusively in the 30 °C acclimation group (N = 10), thus providing an opportunity to compare metabolic recovery profiles of fish that ultimately survived versus those that died during subsequent days (Fig. 1C). Fish suffering delayed mortality were characterised by a prolonged metabolic recovery in comparison with surviving fish, although this was largely driven by the fact that delayed mortality was exclusively linked with high exercise performance scores and thus a larger ‘oxygen debt’ acquired during the fishing simulation (Figs 1C and 2A).

We tested the hypothesis that the temperature-dependence of mortality following the fishing simulation was governed by oxygen transport capacity, as would be predicted by OCLTT. In contrast to this hypothesis, fish from the delayed mortality group (typically 30 °C) and short-term mortality group (typically 33 °C) achieved the same MMR as the survivors within the same temperature (Table 2). Nevertheless, MMR of survivors was higher at 30 °C (4.25 ± 0.13 mg min^−1^ kg^−1^) than at any of the other three temperatures (24 °C: 3.54 ± 0.11 mg min^−1^ kg^−1^ (Holm-Sidak post-hoc test: P = 0.001); 27 °C: 3.52 ± 0.12 mg min^−1^ kg^−1^ (P = 0.002); 33 °C: 3.50 ± 0.23 mg min^−1^ kg^−1^ (P = 0.019)) (Table 2). The combined effects of temperature on RMR and MMR translated to a higher aerobic scope of survivors at 30 °C (3.13 ± 0.11 mg min^−1^ kg^−1^) compared with 27 °C (2.45 ± 0.11 mg min^−1^ kg^−1^; P < 0.001) and 33 °C (2.06 ± 0.19 mg min^−1^ kg^−1^; P < 0.001), but not compared with 24 °C (2.81 ± 0.11 mg min^−1^ kg^−1^; P = 0.107). There was some indication that fish that ultimately suffered delayed mortality did not return to the same level of RMR as fish that survived (orange circles in Fig. 1C), although this pattern was not consistent (cf., Fig. 1B,C inset).

## Discussion

### Post-exercise recovery in a warming world

The present study highlights a mechanism by which climate warming may interact with exhaustive exercise stress (e.g., during catch-and-release of fish) to drive fishing-induced selection. We found a continuum of phenotypes in the propensity for burst exercise in coral grouper, with dramatic consequences for the survival of individuals undertaking high levels of burst swimming at the two warmest acclimation temperatures (30 and 33 °C). Interestingly, the rate of mortality was dichotomous between these two temperatures, whereby mortality at the current summer maximum temperature (30 °C) typically occurred 3–13 d after the exercise challenge, while mortality at the projected summer temperature for 2100 (33 °C) occurred within 4.8 ± 1.1 h after exercise (Figs 2B and 3B). Given the level of mortality observed at 30 °C, and the single mortality observed in an exceptionally high-performance fish at 27 °C (Figs 2A and 3B), it is possible that present-day summer temperatures are already interacting with fishing-related exertion to select against coral grouper with high-performance phenotypes. While links between temperature and fishing-related mortality have been documented previously^22^, the present study identifies intraspecific traits that influence how the structure of within-population phenotypes may be altered by contemporary fishing practices.

The vast difference in exercise propensity between individuals was unexpected prior to the commencement of the research program, as it is known that all coral grouper exhibit at least some level of burst swimming when they are hooked during a real fishing encounter (A. J. Tobin, pers. comm. [commercial fishing licence holder]). Nevertheless, the exercise performance scores exhibited repeatability within individuals (Table 1), providing evidence that our approaches were robust. The fish used in this study were caught from the reef by experienced commercial fishers using heavy fishing gear, and were brought aboard the fishing vessel without delay (typically <15 s). This contrasts with the situation that can occur in some circumstances, particularly in recreational fisheries, where fish can intentionally or unintentionally be subjected to several minutes of exercise and air exposure. While we do not have information on individual ‘fight intensities’ during the original fish capture to link with subsequent exercise performance scores during the fishing simulations, it may be reasonable to expect that the two are correlated. Testing this idea would be a fruitful direction for future research (e.g., ref. 41), but experimental manipulations of ‘fight time’ can be challenging on coral reefs because of the high risks of coral entanglement and shark predation. In any event, if intense exercise is uniformly higher in real fishing encounters than during our lab-based exercise challenges, then the percentage of mortalities documented in the present study may underestimate the likely mortalities during warm periods in the natural environment resulting from inexperienced fishers and long fight times.

While vigorous struggling and heightened burst swimming capacity would likely be advantageous for brief periods in the natural environment to avoid predators and capture prey, we have shown here that the propensity to burst exercise can be maladaptive in the context of contemporary fishing encounters. It is possible to draw parallels with the situation that occurs with large, pelagic fishes in the open ocean – sharks, billfishes and tunas are capable of significant fight times (e.g., >30 h) before they are landed, and post-release mortality can be substantial^42^,^43^,^44^,^45^,^46^. The physiological or biochemical mechanisms causing mortality in coral grouper at high temperatures in the present study may be similar to those causing mortality in large pelagic fishes.

### Mechanisms of mortality after exercise

Despite more than 75 years of scientific interest^47^,^48^, the mechanisms responsible for fish mortality following intense exercise remain poorly understood. Early investigations hypothesised that mortality may be associated with blood acidosis, whereby critically high levels of lactic acid liberated from muscle glycogen diffuse into the blood during exhaustive exercise^48^,^49^,^50^. A subsequent empirical test challenged this hypothesis^30^, instead suggesting that the key toxic event could be *intracellular* acidosis due to the accumulation of lactic acid and an unidentified anion in the intracellular compartment of white (skeletal) muscle cells.

While the responsible mechanisms remain to be elucidated, the present study contributes to the investigation by demonstrating for the first time that mortality in coral grouper is linked with phenotypic variation in the propensity to exercise exhaustively. Moreover, the present study reveals a clear interaction between exercise and temperature, whereby intense exercise alone is rarely lethal without the compounding effects of thermal stress (Fig. 3). Similar interactions between temperature and fishing-related stressors have been reported previously (ref. 22 and references within), suggesting that this may be an important driver of fishing-induced selection/evolution on a global scale.

Impairment of post-exercise maximum oxygen transport capacity (i.e., MMR) does not appear to be a driver of pending mortality (Fig. 1C,D, Table 2), however there may be a threshold post-exercise ‘physiological debt’ beyond which homeostasis cannot be re-established (Fig. 1). That is, the major disruption to physiological homeostasis at high temperatures and extreme exercise levels may result in irreversible damage to cell functioning that leads to mortality within hours (33 °C) or days (30 °C). Our findings suggest that the underlying mechanisms of mortality place major challenges on cellular functions that call upon significant oxygen and energy investment in an effort to regain homeostasis. Indeed, the ‘oxygen debt’ (or excess post-exercise oxygen consumption [EPOC]) illustrated in Fig. 1 consists of a range of energy-demanding processes, such as restoration and balancing of tissue and cellular stores of oxygen, high-energy phosphates, metabolites and ions^15^. Our findings do not invalidate the previous suggestion that mortality may be associated with an inability to regain intracellular pH balance^30^, but they do indicate that delayed mortality can occur many days after metabolic recovery appears complete (Figs 1 and 2A). Regardless of the mechanisms involved, our findings have obvious implications for the sustainable management of coral grouper in the Indo-Pacific, both during current summer periods and with future climate warming.

### Ecological and management implications

The present study shows that fishing-related exercise has the potential to act as a significant selection pressure on specific phenotypes within fished populations, more-so than any natural stressor that is likely to occur without anthropogenic influence. Applying our findings to the natural environment, a disproportionate decrease in the number of high-performance coral grouper in the Indo-Pacific is likely to have impacts on ecosystem dynamics. For example, the gradient in exercise performance scores in coral grouper may be comparable to the bold-shy continuum that is well-documented in other species^51^,^52^,^53^. These continuums could translate to intraspecific niche separation, whereby individuals at extreme ends of the continuum may have distinct functional roles in predator-prey and ecosystem dynamics^54^,^55^,^56^. Indeed, bold or high-performance individuals may have a greater propensity to locate prey across broader spatial scales, whereas shy or low-performance individuals may remain within smaller home-ranges and rely exclusively on ambush rather than roaming foraging tactics^54^,^57^. A disproportionate reduction in high-performance coral grouper in the Indo-Pacific may reduce the existing diversity in home-range size across individuals^39^ and modify inter- and intraspecific trophic interactions^58^,^59^,^60^. While these ideas would be technically challenging to investigate, they would broaden our understanding of trophic dynamics beyond the interspecific level.

Fishing pressure and other human influences are widely-recognised issues in coastal marine ecosystems^61^, such that marine protected areas (MPAs) have been introduced in systems such as the GBR to help mitigate negative consequences. When compliance is strong, MPAs can bolster fish biomass and biodiversity^62^,^63^. The present study illustrates the importance of retaining protected areas in which catch-and-release fishing is prohibited, rather than implementing size limits or catch quotas that do not eliminate capture-related stresses. This study also suggests that phenotypic diversity in coral grouper populations may be bolstered by temporary fishing closures when water temperatures exceed a threshold level, as is the case in some salmonid fisheries^64^,^65^. This may represent a more agile management strategy that avoids many of the complications associated with establishing and policing MPAs. In any event, we show that fishing-induced exercise stress can interact with current-day summer temperatures to drive the selection of particular phenotypes, and additionally we show that these impacts will be exacerbated as the climate continues to warm throughout this century and beyond. This new knowledge should be integrated into management plans (e.g., ref. 66) to help conserve the phenotypic diversity of important fisheries species and reduce the potential for fishing-induced selection.

## Methods

### Animals and holding conditions

This research was conducted in accordance with all relevant regulations and with the approval of the Animal Ethics Committee of James Cook University (A1723). Wild coral grouper (*Plectropomus leopardus*; N=168) were caught on the Great Barrier Reef (GBR) during June 2012 using baited hook-and-line from aboard commercial fishing vessels. Commercial fishing gears and methods meant that fight times were generally short (typically <15 s) and captured fish had minimal air exposure (typically <5 s) before being released (if undersized) or retained in on-board holding tanks (live wells). Approximately half of the fish (N = 83; mean body mass (*M*_b_) ± SE = 1.48 ± 0.12 kg; total length (TL) = 47.0 ± 1.0 cm) were obtained near Heron Island at the southern end of the GBR, while the others (N = 85; *M*_b_ = 1.28 ± 0.06 kg; TL = 46.1 ± 0.6 cm) were obtained near Princess Charlotte Bay in the northern GBR. Water temperatures at the time of capture were approximately 22 °C and 25 °C for Heron Island and Princess Charlotte Bay regions, respectively. Coral grouper are protogynous hermaphrodites that generally change sex from female to male at a TL between 25 and 64 cm. Thus, both sexes were represented in this study, but underdeveloped gonads precluded the differentiation of sexes herein.

Fish were transported by boat (live wells) and then by road (oxygenated holding tanks) to the Marine and Aquaculture Research Facilities Unit (MARFU), James Cook University, Townsville, Australia. They were equipped with two colour-coded and individually identifiable T-bar tags in the dorsal tissue prior to being allocated to one of eight 2,000 L holding tanks receiving flow-through water at 26 °C (N = 21 fish per tank). Vigorous aeration was provided to all tanks using ceramic diffusers connected to a central air compressor system. Dissolved oxygen remained >90% of air saturation at all times. Fish were allowed to adjust to the holding tanks for 3–4 weeks prior to any further disturbances to ensure that most individuals had commenced feeding and were visibly healthy. No mortalities occurred during this time. Food (thawed pinkies, *Nemipterus* spp.) was provided at satiation levels every second day.

### Thermal acclimation

Once fish had adjusted to the holding tanks and most were feeding well, each tank was randomly assigned to one of four temperature treatments (24, 27, 30 or 33 °C; two tanks per temperature). The temperatures were selected to cover most of the natural range experienced by both populations (annual monthly mean ranges for Heron Island and Princess Charlotte Bay are approximately 21–27 °C and 24–29 °C, respectively) as well as the high temperature of 33 °C that is predicted to occur more frequently by the year 2100 (i.e., ~4 °C above current mean monthly maximum [~2 °C above extreme daily maximum] for the northern GBR)^1^. Tank temperatures were achieved by heating or cooling the tanks at 0.5 °C d^−1^ using heaters and chilling units as necessary. Once the experimental temperatures had been achieved, the fish were given 2–3 weeks before a subset of fish (fasted for 48 h) from each of 24 °C (N = 16) and 30 °C (N = 16) were selected to undergo an initial catch-and-release fishing treatment with subsequent recovery in respirometers (these subsets of fish were termed ‘early acclimation’; see below). These initial trials were conducted to compare against later experiments in order to gain an understanding of the temporal patterns of metabolic acclimation and the repeatability of burst swimming performance. The 32 fish were returned to their respective holding tanks following respirometry, and all individuals were given at least another 3 weeks to thermally acclimate while satiation feeding continued every second day. Individual feeding rates were recorded to pinpoint any individuals that were not regularly taking food. Individuals were not included in subsequent experiments if they did not commence a healthy level of feeding and subsequently suffered appreciable weight loss during the thermal acclimation period (bringing total to N = 132 herein). Sample sizes for all subsequent experiments were 39 (24 °C), 32 (27 °C), 35 (30 °C), and 26 (33 °C) (Table 2). There were no differences in mean fish mass across treatment groups (ANOVA, *F*_(3,128)_ = 0.143, P = 0.934).

### Catch-and-release fishing simulation

An exhaustive exercise protocol was designed to simulate a catch-and-release fishing encounter. For the early-acclimation trials (N = 32), and following the ~6-week thermal acclimation (N = 132; herein termed ‘full-acclimation’), fish were fasted for 48 h before being individually dip-netted from their holding tank and placed into a round exercise tank (~300 L, diameter 1 m, water depth 0.4 m) that was maintained at the corresponding acclimation temperature. The fish was then encouraged to burst swim by two experimenters who splashed the surface of the water and rapidly tapped the tail of the fish to encourage maximal exercise^49^. The exercise protocol continued for 3 min and the tail taps transitioned to tail grabs as necessary to ensure the fish continued to be stimulated for the duration of the trial. At the end of 3 min, the fish was dip-netted and held in air for 1 min to simulate the post-capture period in a catch-and-release fishery (e.g., for hook removal, admiration, photographs, etc) before being placed into a respirometer at the corresponding experimental temperature to quantify the entire post-exercise metabolic recovery period (details below). The exercise and air exposure times used here are likely to be more similar to situations in the recreational sector than the commercial sector, but our findings are broadly applicable to both (see Discussion).

Earlier experiments on a different subset of coral grouper revealed a large diversity in the response of individual fish to the exercise protocol. Thus, in the present study we used a burst exercise scoring system to grade the effort of each fish during the trials, where the scoring ranged from 1 (poor) to 5.5 (exceptional) in increments of 0.5. A score of 1 was given to a fish that was lethargic, did not perform any significant bursting activity, and generally allowed the experimenters to grab the tail with little attempt to escape. A score of 5 was given to a fish that was extremely responsive to the experimenters and underwent near-continuous bursting around the circular tank for 90–120 s before becoming lethargic, and even then remained responsive to subsequent tail grabs for the remainder of the 3 min period. A score of 5.5 was given for only one individual (at 27 °C ) that maintained a level of burst performance that was not witnessed in any other individual (i.e., continuous bursting around the tank for nearly the entire 3 min protocol). Experimenters during the full-acclimation trials were blind to the exercise performance scores obtained by the subset of 32 individuals used in the early-acclimation trials.

### Respirometry

Following the 3 min exercise protocol and 1 min air exposure (during which time each fish was measured and weighed), each fish was placed immediately into a cylindrical respirometer (23.5 cm diameter, 68 cm length, 30 L volume) at the treatment temperature and measurements of oxygen consumption rates (O_2_) commenced within 20 s (one larger respirometer was occasionally used to accommodate large individuals: 23.5 cm diameter, 100 cm length, 44 L volume). Eight intermittent flow-through respirometers were used in parallel throughout the experimental period. The design of the respirometers and the respirometry protocol followed best practices outlined previously^33^. Briefly, each respirometer had a closed-circuit recirculation loop that ensured homogenous oxygen levels throughout the respirometer at all times, and the respirometers were connected to an automated flush pump that flushed the respirometers with air-saturated water for 5–8 min in every 10–15 min period (flush duration and frequency increased at higher temperatures to account for elevated fish metabolism). Oxygen levels within the respirometers were monitored (at 0.5 Hz) within the recirculation loop at all times using a fibre-optic system and contactless oxygen sensor spots (FireSting O_2_, PyroScience, Germany), and O_2_ was calculated from the decline in oxygen concentration in the respirometers between flush cycles. Oxygen levels within the respirometers remained above 80% air saturation at all times. Fish remained in the respirometers for ~20 h following the catch-and-release simulation in order to track the entire period of metabolic recovery from the elevated levels immediately after the simulation through to the point where O_2_ had plateaued at resting levels (see *Data analysis and statistics*). Fish were returned to their respective holding tanks following respirometry. Respirometers were cleaned regularly to ensure that background microbial respiration remained negligible.

### Post-treatment survival

There was significant mortality associated with the fisheries capture treatment, and so individuals were categorised into three distinct groups: (1) fish that died in the respirometers post-treatment (i.e., within ~20 h) were considered to be ‘short-term mortalities’; (2) fish that died within 13 d post-treatment (all were between 3–13 d) were considered to be ‘delayed mortalities’; and (3) fish that survived greater than 13 d (marking the end of the study) were considered to be ‘survivors’.

### Data analysis and statistics

Metabolic data were analysed after importing the text file from the FireSting O_2_ software into LabChart 7 (ADInstruments Pty Ltd, Bella Vista, New South Wales, Australia). Linear regressions between water oxygen concentration and time were made for each measurement period and the slopes derived from the regressions were used to calculate mass-specific aerobic metabolic rate (mg O_2_ min^−1^ kg body mass^−1^) after accounting for the volume of the respirometer (minus the volume of the fish).

Maximum metabolic rate (MMR) was determined as the highest O_2_ value occurring in any 3 min period throughout the ~20 h respirometry protocol, which almost always occurred within the first hour after the fishing simulation. Post-exercise resting metabolic rate (RMR) was determined by first taking the mean of the lowest 10% of O_2_ measurements over the 20 h respirometry period, removing outliers (±2 SD from the mean; no more than two data points were identified as outliers for any fish), and then calculating the mean of the remaining measurements. Periods of elevated metabolism associated with spontaneous activity in the respirometers were excluded from analyses of RMR and metabolic recovery. Metabolic recovery duration was calculated for each individual as the time taken post-treatment for three consecutive O_2_ measurements to fall to within ±1 SE of that individual’s RMR value (recovery duration was calculated to the first of these three points).

Statistical analyses were performed using R software and SigmaPlot 11 (Systat Software Inc., San Jose, CA, USA). Prior to statistical analyses, metabolic data and recovery durations were log-transformed where necessary to satisfy assumptions of normality, while exercise performance scores were converted to proportional data and logit-transformed. Differences between southern and northern populations were assessed for all measured variables (RMR, MMR, exercise performance score, and recovery duration) using two-way ANOVAs with acclimation temperature and population as factors, and including the temperature*population interaction. Only ‘survivors’ were used to compare across populations and temperatures (unless otherwise stated) to maintain consistency and to ensure complete datasets for each fish (e.g., RMR was not measureable in ‘short-term mortalities’). No significant differences between populations were detected in any test (P range 0.165–0.642), so populations were pooled for subsequent analyses. One-way ANCOVA was used to test for differences in the relationship between exercise performance score (factor) and recovery duration (dependent variable) controlling for acclimation temperature (covariate). ANOVA and t-tests were used where indicated to test for differences between acclimation temperatures. The overall level of significance was P < 0.05 but the critical level was adjusted for multiple comparisons where necessary using Holm-Sidak post-hoc tests. Values are presented as means ± SE unless otherwise indicated.

## Additional Information

**How to cite this article**: Clark, T. D. *et al*. Rising temperatures may drive fishing-induced selection of low-performance phenotypes. *Sci. Rep.* **7**, 40571; doi: 10.1038/srep40571 (2017).

**Publisher's note:** Springer Nature remains neutral with regard to jurisdictional claims in published maps and institutional affiliations.

## Figures and Tables

**Table 1:** Body mass (*M*_b_), exercise performance scores (EPS), post-exercise fate, resting metabolic rate (RMR), maximum metabolic rate (MMR) and aerobic scope (AS) of individual coral grouper tested within 2–3 weeks of reaching their treatment temperature (early-acclimated) and subsequently following ~6 weeks of thermal acclimation (fully-acclimated).

Fish#	Early-acclimated						Fully-acclimated					
	*M*_b_	EPS	Fate	RMR	MMR	AS	*M*_b_	EPS	Fate	RMR	MMR	AS
**24 °C**												
1	1.34	4.5	survived	0.74	2.76	2.02	1.38	4.0	survived	0.80	3.66	2.86
2	0.89	1.0	survived	0.64	4.56	3.93	0.93	1.5	survived	0.63	4.12	3.49
3	0.78	2.0	survived	0.90	3.55	2.65	0.72	1.5	survived	0.68	2.95	2.27
4	0.97	3.5	survived	0.86	4.34	3.48	1.00	1.5	survived	0.88	4.11	3.23
5	0.63	4.5	survived	0.93	4.02	3.09	0.69	4.5	survived	0.45	3.69	3.23
6	0.97	4.5	survived	1.43	4.21	2.78	1.05	2.5	survived	0.74	3.99	3.25
7	1.04	5.0	survived	1.38	2.89	1.51	1.00	5.0	survived	1.14	3.07	1.92
8	0.98	1.5	survived	0.92	4.14	3.22	0.93	1.5	survived	0.84	4.29	3.45
9	0.88	1.0	survived	0.88	3.87	2.99	0.88	1.0	survived	0.51	3.50	2.98
10	0.95	3.0	survived	0.93	4.82	3.89	0.98	1.5	survived	0.86	4.35	3.49
11	0.78	1.5	survived	0.69	4.30	3.60	0.76	1.5	survived	0.65	4.03	3.38
12	1.33	2.0	survived	0.89	4.80	3.91	1.30	2.5	survived	0.90	3.90	3.00
13	1.29	1.0	survived	0.76	3.44	2.69	1.28	1.5	survived	0.63	3.70	3.07
14	1.28	1.0	survived	1.27	5.33	4.06	1.24	1.5	survived	0.70	3.43	2.74
15	0.72	1.5	survived	0.82	4.27	3.45	0.72	1.5	survived	0.54	4.59	4.05
16	0.97	1.5	survived	0.86	3.66	2.80	0.97	1.5	survived	0.50	3.48	2.98
***Mean***	***0.99*** ± ***0.05***	***2.4*** ± ***0.4***	—	***0.93*** ± ***0.06***	***4.06*** ± ***0.17***	***3.13*** ± ***0.18***	***0.99*** ± ***0.05***	***2.2*** ± ***0.3***	—	***0.72*** ± 0.05*	***3.80*** ± ***0.11***	***3.09*** ± ***0.13***
**30 °C**												
1	1.29	4.5	del. mort.	1.57	3.73	2.16	—	—	—	—	—	—
2	1.29	2.5	survived	1.05	4.56	3.51	1.29	1.5	survived	1.21	4.70	3.48
3	0.87	2.0	survived	1.30	4.71	3.41	0.87	1.0	survived	1.04	4.13	3.09
4	0.96	5.0	del. mort.	1.96	3.96	2.00	—	—	—	—	—	—
5	0.89	4.0	survived	1.48	4.12	2.65	0.84	4.5	survived	1.34	4.48	3.14
6	1.26	2.0	survived	1.89	7.19	5.30	1.27	1.5	survived	1.17	5.11	3.93
7	0.87	3.5	survived	1.41	4.30	2.89	0.85	2.5	survived	1.25	4.58	3.33
8	1.71	3.5	survived	1.60	3.93	2.33	1.45	2.5	survived	1.00	4.59	3.59
9	0.76	5.0	del. mort.	1.94	4.05	2.11	—	—	—	—	—	—
10	0.80	1.5	survived	1.23	5.24	4.00	0.84	2.5	survived	1.26	4.69	3.43
11	0.72	4.0	del. mort.	1.85	3.99	2.14	—	—	—	—	—	—
12	1.00	1.0	survived	1.39	3.91	2.51	1.16	1.5	survived	1.30	4.84	3.53
13	1.17	1.5	survived	1.35	4.66	3.31	1.20	2.5	survived	1.20	4.78	3.58
14	0.89	1.5	survived	1.87	7.07	5.21	0.95	2.5	survived	1.07	5.15	4.08
15	0.96	1.5	survived	1.36	4.52	3.16	0.98	1.5	survived	1.58	5.07	3.49
16	0.69	2.0	survived	1.12	4.31	3.19	0.74	4.5	del. mort.	1.43	4.22	2.79
***Mean***	***1.01*** ± ***0.07***	***2.8*** ± ***0.3***	—	***1.53*** ± ***0.08***	***4.64*** ± ***0.26***	***3.12*** ± ***0.25***	***1.03*** ± ***0.07***	***2.4*** ± ***0.3***	—	***1.24*** ± ***0.05***	***4.69*** ± ***0.09***	***3.46*** ± ***0.10***

*significantly lower than the corresponding value in the early-acclimation group (paired t-tests on metabolic parameters −24 °C RMR: t_(15)_ = 3.801, **P** = **0.002**; 24 °C MMR: t_(15)_ = 1.673, P = 0.115; 24 °C AS: t_(15)_ = 0.299, P = 0.769; 30 °C RMR: t_(11)_ = 1.762, P = 0.106; 30 °C MMR: t_(11)_ = 0.656, P = 0.525; 30 °C AS: t_(11)_=0.004, P = 0.997).

These duplicate tests were performed on a subset of 16 individuals from each of the 24 °C and 30 °C treatment groups.

**Table 2:** Measured variables for fully thermally acclimated (~6 weeks) coral grouper at the time of the fishing capture simulation, where fish are divided based on their temperature treatment group and their fate (short-term mortality, delayed mortality, survived).

	24 °C			27 °C			30 °C			33 °C		
	short-term mort.	del. mort.	survive	short-term mort.	del. mort.	survive	short-term mort.	del. mort.	survive	short-term mort.	del. mort.	survive
N	0	0	39	0	1	31	3	6	26	15	0	11
*M*_b_ (kg)	—	—	1.3 ± 0.1	—	1.1	1.4 ± 0.2	1.4 ± 0.6	1.0 ± 0.1	1.5 ± 0.1	1.0 ± 0.1	—	1.7 ± 0.3
RMR (mg min^−1^ kg^−1^)	—	—	0.73 ± 0.03^A^	—	1.44	1.07 ± 0.06^B^	—	1.64 ± 0.06^#^	1.12 ± 0.04^B^	—	—	1.45 ± 0.06 ^C^
MMR (mg min^−1^ kg^−1^)	—	—	3.54 ± 0.11 ^A^	—	3.41	3.52 ± 0.12 ^A^	3.72 ± 0.30	4.00 ± 0.18	4.25 ± 0.13^B^	3.60 ± 0.15	—	3.50 ± 0.23 ^A^
AS (mg min^−1^ kg^−1^)	—	—	2.81 ± 0.11^AB^	—	1.97	2.45 ± 0.11^BC^	—	2.36 ± 0.18^#^	3.13 ± 0.11^A^	—	—	2.06 ± 0.19 ^C^
FAS	—	—	5.12 ± 0.24^B^	—	2.37	3.51 ± 0.17 ^A^	—	2.50 ± 0.14^#^	3.87 ± 0.13 ^A^	—	—	2.42 ± 0.12^C^

Resting metabolic rate (RMR) was not measureable in short-term mortalities, and thus aerobic scope (AS) and factorial aerobic scope (FAS) could not be calculated. MMR is maximum metabolic rate. Dissimilar letters indicate results of one-way ANOVAs, where metabolic parameters were compared across temperatures within a fate grouping. ^#^significantly different from corresponding value for survivors within 30 °C. There was no difference in MMR between short-term mortalities and survivors at 33 °C. Any groups containing sample sizes <6 were not included in statistical comparisons.
